# Unusual Presentation of Giant Cell Arteritis With Localised Temporal Artery Aneurysm

**DOI:** 10.7759/cureus.95624

**Published:** 2025-10-28

**Authors:** Syed A Ahmad, Niazi Khairi, Meeran Hussain, Mark Lloyd

**Affiliations:** 1 General Medicine, Frimley Park Hospital, Camberley, GBR; 2 Rheumatology, Frimley Health NHS Foundation Trust, Camberley, GBR

**Keywords:** colored flow doppler ultrasound, fdg pet/ct scan, giant cell arteritis (gca), temporal aortic aneurysm, temporal artertitis, vasculitis

## Abstract

Giant cell arteritis (GCA) is a granulomatous vasculitis of medium and large-sized arteries occurring in older adults. Skip lesions are well described, but to our knowledge, localised aneurysm formation in the context of normocalcaemic, non-progressive GCA has not been reported. We report a case of a 74-year-old male who presented with painless left-sided temporal artery swelling, with an aneurysm and halo sign on ultrasound Doppler (US Doppler). Inflammatory markers were normal. Biopsy of the affected segment confirmed granulomatous inflammation with giant cells. Without treatment, he remained well with subsequent normal inflammatory markers and the US Doppler and positron emission tomography (PET)-CT scan showing no progression of the disease.

This case illustrates the wide spectrum of giant cell arteritis, with the inflammation here being restricted to a focal segment of the superficial temporal artery with normal inflammatory markers and no apparent progression or similar isolated pockets in other areas. The temporal artery aneurysm is also a complication that has not been seen before in the context of GCA. This highlights the importance of considering a diagnosis of GCA, even in the absence of a classical presentation and laboratory findings, based on a potential complication that can help uncover the underlying issue.

## Introduction

Giant cell arteritis (GCA) is a chronic granulomatous vasculitis that primarily affects the walls of medium and large-sized arteries in older adults [1], most notably the temporal arteries and other extracranial branches of the carotid arteries, as well as the aorta and its major branches. It is the most common vasculitis in individuals over 50 years of age, with an incidence of 10:100,000 in this population [2]. It carries a significant risk of complications, including severe complications such as irreversible visual loss, stroke, myocardial infarction, aortic dissection, and aneurysm [3].

GCA typically presents with cranial manifestations, such as headache, scalp tenderness, jaw claudication, and visual disturbance, but may also cause systemic symptoms, including fever and malaise. The diagnosis of GCA is supported by elevated inflammatory markers such as C-reactive protein (CRP) and erythrocyte sedimentation rate (ESR), although normal values do not exclude the disease. There are also some scoring systems in use in clinical research, such as the 2022 American College of Rheumatology European Alliance of Associations for Rheumatology (ACR/EULAR)-endorsed classification criteria for GCA [4]; however, in clinical practice, the diagnosis is usually confirmed through histopathology or imaging [5]. In the UK, ultrasound Doppler is generally the first-line investigation; however, advanced imaging such as positron emission tomography (PET)-CT or magnetic resonance angiography (MRA) may be considered to assess for large-vessel involvement. Temporal artery biopsy is still the gold standard for confirmation, but it is limited by false negatives and increasing difficulty in finding surgeons with the skill to perform the biopsy. High-dose glucocorticoids remain the cornerstone of initial treatment [5].

## Case presentation

A 74-year-old white British male, previously fit and well apart from a well-controlled bilateral primary open-angle glaucoma, presented to his general practitioner in December 2023 with a painless, non-tender, and pulsatile mass over the left temple, which had enlarged over the last two months. He was a lifelong non-smoker with no history of hypertension or ischaemic heart disease. On initial assessment, he complained of dizziness and headaches, although no focal neurology was present. His vitals were stable.

An ultrasound Doppler (US Doppler) of the left superficial temporal artery was performed in January 2024 using a portable scanner, which showed a circumferential hypoechoic wall thickening involving the frontal branch, measuring 1 mm in thickness and extending over 15 mm in length. These findings were suggestive of temporal arteritis.

This was discussed with a rheumatology consultant at Frimley Park Hospital, who advised commencing him on prednisolone 40 mg OD and to send off his inflammatory markers, which were normal with a C-reactive protein (CRP) <1.0 (0-5 mg/L) and erythrocyte sedimentation rate (ESR) of 8 (0-22 mm/hour).

A repeat US Doppler of the affected segment was also performed after one week of prednisolone in the hospital to assess the response to steroids in the absence of positive inflammatory markers, which showed a patent vessel, however at the area of the visible lump, the frontal branch appeared aneurysmal measuring 2.5 mm (max AP) with intimal thickening of 0.6 mm demonstrating the 'halo' sign on cross-section which is classical for GCA.

The patient, however, was asymptomatic at this time. It appeared that the headache and dizziness that he had initially presented with had spontaneously resolved. His prednisolone was stopped at this point because of the atypical features and was not subsequently given. An alternative diagnosis was thought to be more likely, such as an asymptomatic dilatation of the left temporal artery secondary to a viral illness, given the complete absence of systemic symptoms, normal inflammatory markers, and the atypical finding of an aneurysm, which is not a classic feature of GCA.

In February 2024, a repeat CRP and vasculitic screen was sent, as seen in Table 1.

**Table 1:** Labs show a normal vasculitic screen and CRP levels CRP - C-reactive protein

Test name	Result	Reference range
Complement C3	1.02	0.75 - 1.65 g/L
Complement C4	0.24	0.14 - 0.54 g/L
Rheumatoid factor	< 20.0	0 - 29 IU/ml
Anti-nuclear antibody	0.1	0 - 0.6 Ratio
Myeloperoxidase antibody	< 0.2	0 - 3.4 U/ml
Proteinase 3 antibody	< 0.6	0 - 1.9 U/ml
CRP	< 1.0	0 - 5 mg/L

**Figure 1:**
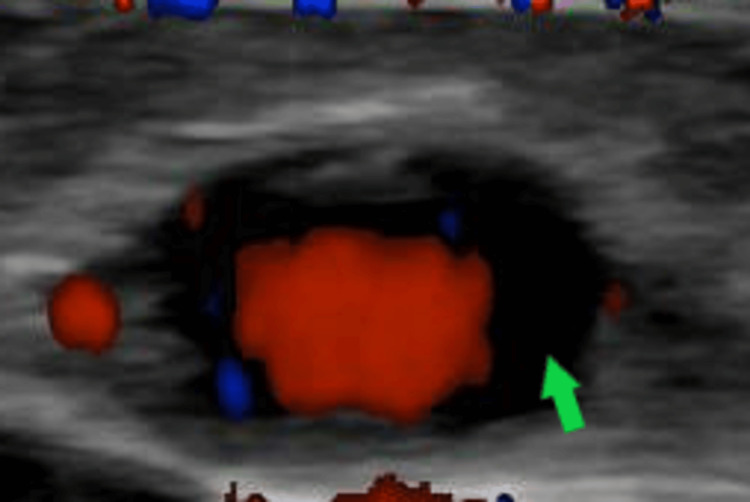
Colour Doppler ultrasound of this patient showing a transverse view of the frontal branch of the left temporal artery which demonstrates a classic "halo sign" representing the inflamed and swollen arterial walls that is a hallmark of GCA GCA - giant cell arteritis

**Figure 2:**
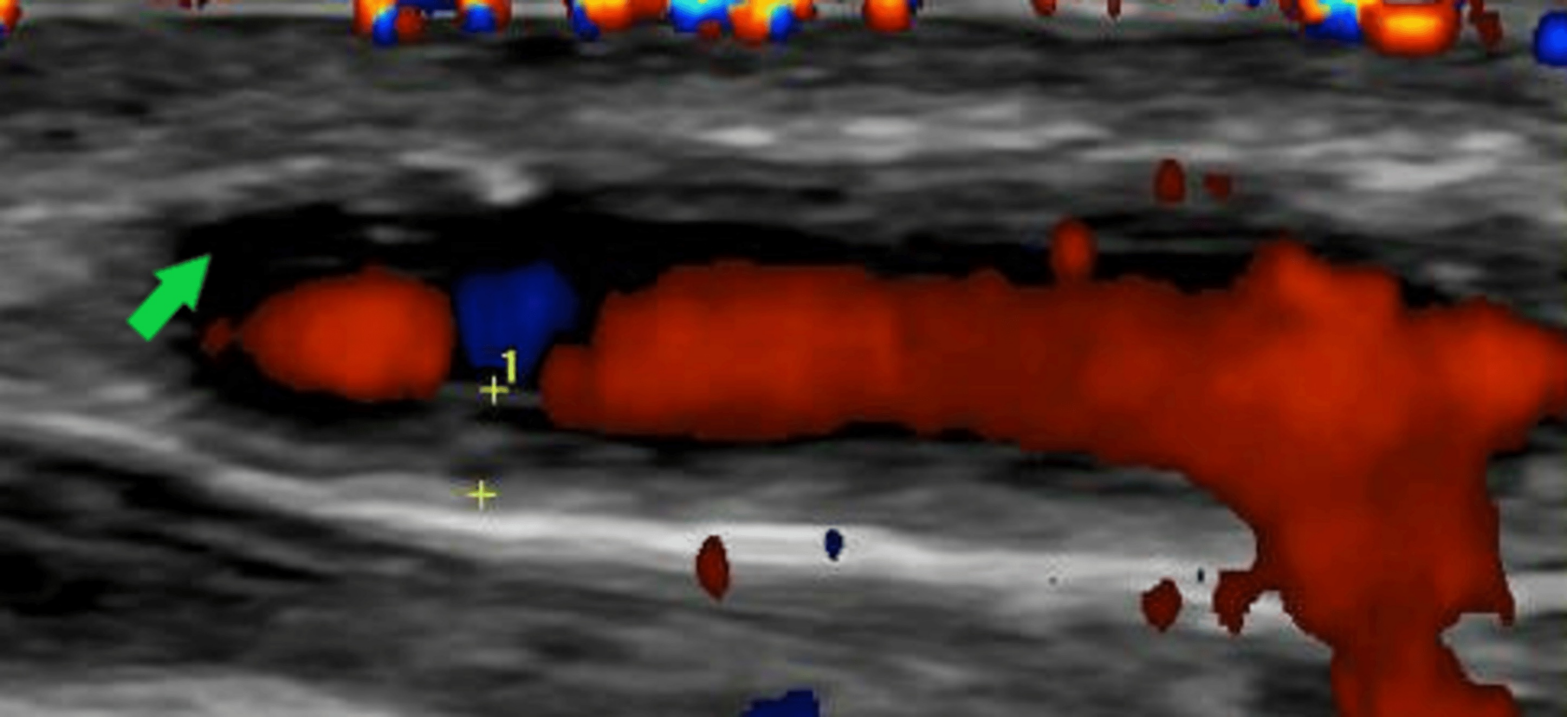
Colour Doppler ultrasound showing a longitudinal view of the frontal branch of the left temporal artery which demonstrates a thickened arterial wall with a narrowed lumen due to the inflamed and swollen arterial walls

A vascular surgery referral was made as a result; however, a repeat US Doppler in April 2024 showed a slight increase in the size of the aneurysm, measuring 2.8 mm (max AP), and with intimal thickening (0.9 mm) and a halo sign as seen in Figure 1 and Figure 2.

Due to this increase in size of the aneurysm, a multidisciplinary decision between rheumatology and vascular surgery was made to perform an excisional biopsy of the aneurysmal segment. In May 2024, the biopsy was performed, which showed histological evidence of GCA with significant transmural lymphohistiocytic inflammation with moderate eosinophilic infiltration, scattered multinucleated giant cells, evidence of vessel wall destruction, and capillary proliferation. 

As he remained otherwise asymptomatic, the patient was then lost to follow-up for over a year before being reviewed again by a Rheumatology Consultant in July 2025. He was well and did not take any steroids during this time. He had a full set of routine bloods, which were unremarkable as seen in Table 2.

**Table 2:** Routine bloods on follow up, all of which are unremarkable eGFR - estimated glomerular filtration rate; CRP - C-reactive protein; WBC - white blood cells

Test name	Result	Reference range
Sodium	141	133 - 146 mmol/L
Potassium	3.9	3.5 - 5.3 mmol/L
Urea	6.3	2.5 - 7.8 mmol/L
Creatinine	77	64 - 104 umol/L
eGFR result/1.73m² (EPI)	85	90 - 120 mL/min
CRP	< 1.0	0 - 5 mg/L
Haemoglobin	150	130 - 180 g/L
WBC	6.4	4 - 11 10⁹/L
Platelets	202	150 - 450 10⁹/L
Total bilirubin	13	0 - 20 umol/L
Alkaline phosphatase	81	30 - 130 U/L
Alanine transaminase	20	0 - 55 U/L
Albumin	40	35 - 50 g/L

**Figure 3:**
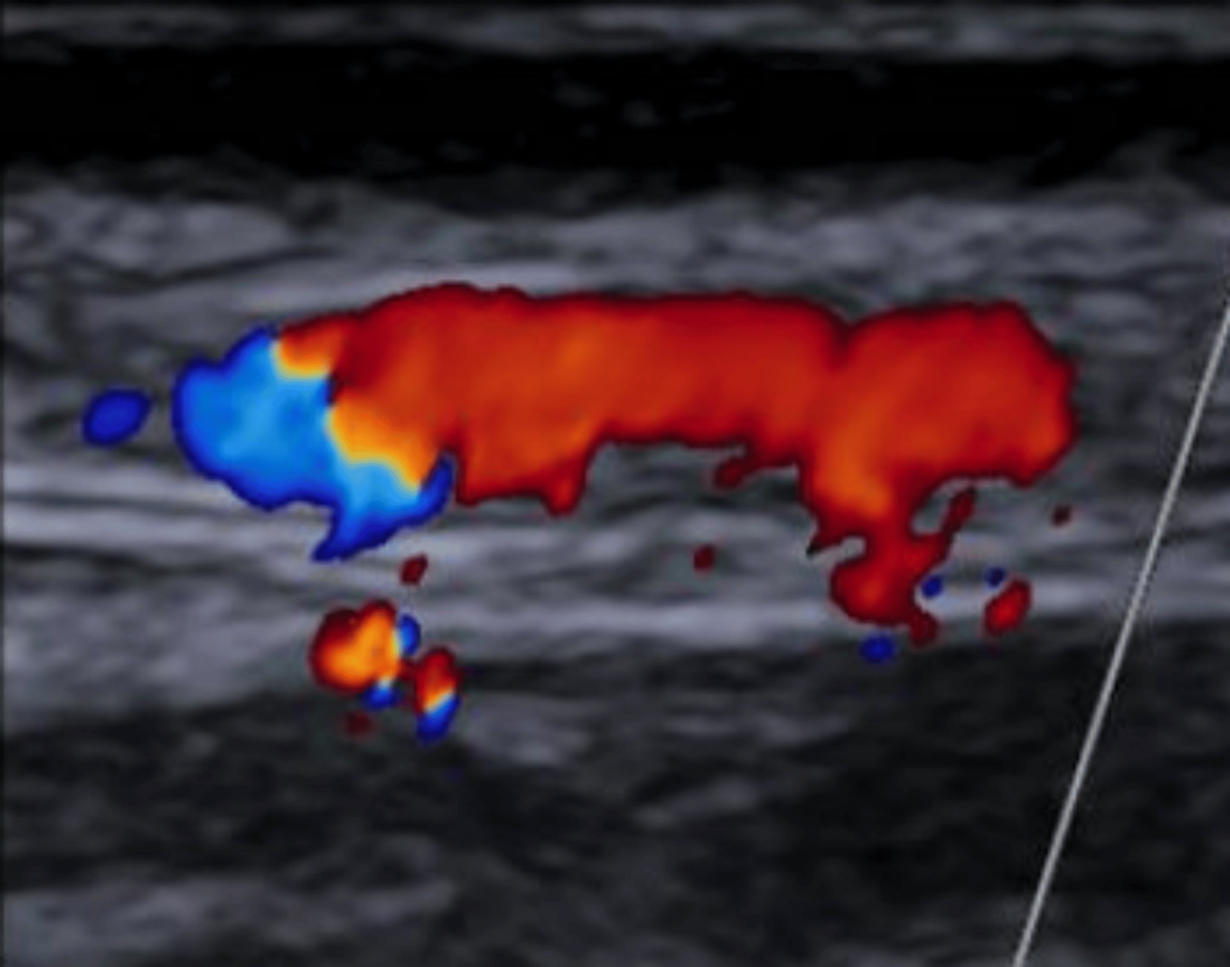
A longitudinal section of his repeat temporal artery colour doppler in July 2025 which shows a patent proximal frontal branch of the temporal artery with no evidence of vessel wall thickening or occluded lumen. The affected part however could not be visualised due to the biopsy.

A further US Doppler scan was performed to exclude further involvement of the cranial or carotid arteries, which demonstrated normal carotid, subclavian, and axillary arteries bilaterally. Both temporal arteries were also patent, including the proximal left frontal branch of the temporal artery as seen in Figure 3, although the left frontal branch was not visualised distal to the excision site.

**Figure 4:**
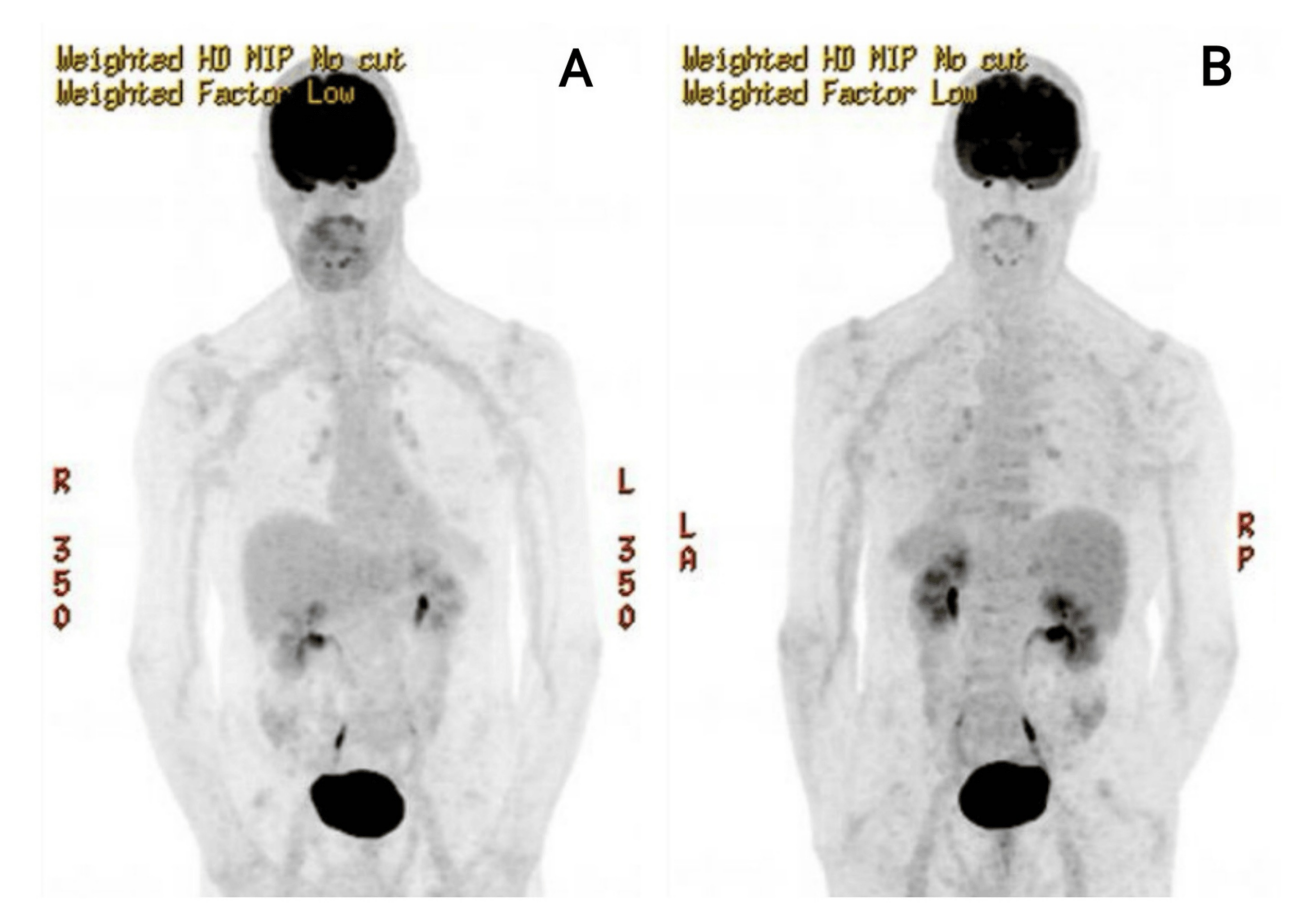
An FDG PET CT scan of the anterior (A) and posterior (B) body from the head to the pelvis, showing a normal radionucleotide uptake and no abnormal areas of inflammation FDG - fluorodeoxyglucose; PET - positron emission tomography

An 18F-FDG PET-CT was also performed to look for inflammation in other arteries and exclude systemic large-vessel involvement, showing mildly increased FDG uptake in the thoracic aorta (SUVmax 1.7, within background range), felt not to be consistent with active large-vessel vasculitis. This can be seen in Figure 4.

There was no further workup done, given his normal imaging and blood results, and he remains under the rheumatology team, who will continue to follow him up.

## Discussion

GCA is a systemic vasculitis but can also affect a single blood vessel, with the temporal artery being most commonly affected [6]. However, this case is unusual in that we are not aware of any previous case reports regarding it causing an isolated temporal artery aneurysm. An aneurysm of the superficial temporal artery (STA) itself is a very uncommon event, and even as recently as 2018, less than 400 cases have been reported, with only about 5% being a true aneurysm [7]. Regarding their causality, a recent review of 63 cases of temporal artery aneurysm showed the main trunk and then the anterior temporal artery to be most commonly affected. No cases of GCA were described as causative - atherosclerosis and segmental arterial mediolysis were the commonest causes, present in over 70% of cases [8].

He was also noted to have normal inflammatory markers, CRP, and ESR, with the CRP being normal in multiple blood tests over more than a year, including when he was off the steroids, which would have suppressed inflammation otherwise. ESR, alone, can be normal in up to 15% of GCA cases; however, having both a normal CRP and ESR is very rare, occurring in less than one percent (0.8%) of cases [9].

In addition to this, his PET CT scan was also mostly unremarkable, only showing a mildly increased FDG uptake in the thoracic aorta, which was not thought to be convincing for a metabolically active large-vessel vasculitis. This is unusual as not only does PET CT have a very good sensitivity, 92%, and specificity, 85%, for GCA, it has a very high negative predictive value of 98% [10]. However, as seen in this case, there was no significant abnormality noted.

These unusual features provide a diagnostic challenge and raise the question as to whether there may be an alternative explanation for our patient's presentation. In this regard, however, he had no atherosclerotic risk factors and his biopsy was also typical for GCA, with transmural inflammation being the most common histopathological finding as per a study from 2014 [11], making up 77.5 % of all positive biopsies for an inflamed temporal artery of which the vast majority, 98.4%, were caused by GCA. The other histopathological findings that were present, including lymphohistiocytes and giant cells, further support this diagnosis [4]. Eosinophilic infiltration is a bit less common, present in under 10% of patients with transmural infiltration, but is still seen in GCA and is actually associated with an increased severity of disease [12]. While this makes the benign nature of this presentation even more unusual, this could be explained by there being only moderate infiltration as opposed to severe. Putting together all these histopathological features, the evidence appears to be enough to conclude that this is indeed GCA, although an atypical case.

Aside from the diagnostic challenge, another unusual feature of this case is that the inflammation was entirely localised to a single segment of the frontal branch of the left temporal artery with no other pockets of inflammation identified elsewhere. Having isolated sections of inflammation is not uncommon in GCA, which often has "skip lesions" defined as segments of inflammation separated by non-inflamed sections on the same blood vessel, with an incidence as high as 28% [13]. This, however, represents a 'solitary lesion', distinct from classic 'skip lesions', which are multiple by definition [14]. We have not identified any other case in the available literature describing such a geographically restricted, solitary inflammatory focus in GCA.

This solitary segment of inflammation may help explain the mild symptoms, normal CRP and ESR, and limited extent of disease, but makes assessing the response to treatment more difficult. Regarding imaging, the 2023 EULAR guidelines suggest an initial assessment with a temporal and axillary artery US Doppler, with follow-up imaging being individualised on a case-by-case basis [15]. In this case, a PET CT was deemed appropriate due to the risk of other similar silent inflammatory or aneurysmal changes in the other large vessels of his body, similar to a case where the patient had both an intracranial and extracranial aneurysm associated with GCA [16].

Although his prednisolone was stopped and he remained asymptomatic, the fact that he already presented with one aneurysm is a significant concern, given that the risk of a new aortic aneurysm is approximately twofold in GCA [17]. This concern is underscored by the catastrophic potential of aortic complications, as demonstrated in cases of fatal ruptured aortic dissection [18], highlighting the critical importance of long-term vascular surveillance in GCA patients even with localized presentations.

## Conclusions

We describe a rare case of GCA presenting as a solitary temporal artery aneurysm with normal inflammatory markers and a non-progressive course. This highlights that GCA can manifest as a localized structural complication without systemic signs, necessitating a high index of suspicion for timely diagnosis. This also highlights the importance of reporting new features or complications of a disease, which might help contribute to the existing literature and assist with recognition in the future.

The long-term effects associated with this sort of localised GCA are not yet known, and it would be useful to identify other similar cases and to compare between different presentations. Given that this patient presented with an aneurysm in spite of an otherwise benign appearing disease, it is reasonable to assume that he continues to be at risk of further complications as well. He will continue to be followed up for his symptoms, repeat inflammatory markers, and also further imaging as needed.
